# De novo mutations within metabolism networks of amino acid/protein/energy in Chinese autistic children with intellectual disability

**DOI:** 10.1186/s40246-022-00427-7

**Published:** 2022-11-01

**Authors:** Wen-Xiong Chen, Bin Liu, Lijie Zhou, Xiaoli Xiong, Jie Fu, Zhi-Fang Huang, Ting Tan, Mingxi Tang, Jun Wang, Ya-Ping Tang

**Affiliations:** 1 The Assessment and Intervention Center for Autistic Children, Department of Neurology, Guangzhou Women and Children’s Medical Center, Guangzhou Medical University, Guangzhou, 510623 Guangdong China; 2 Guangzhou Institute of Pediatrics, Guangzhou Women and Children’s Medical Center, Guangzhou Medical University, Guangzhou, 510623 China; 3 Department of Biobank, Shenzhen Baoan Women’s and Children’s Hospital, Jinan University, Shenzhen, 518102 Guangdong China; 4 Department of Pediatric Rehabilitation, Third Affiliated Hospital of Zhengzhou University, Zhengzhou, 450052 China; 5 Department of Pathology, Affiliated Hospital of Southwest Medical University, Luzhou, 646000 Sichuan China; 6 Guangdong Provincial Key Laboratory of Brain Function and Disease, Zhongshan School of Medicine, Sun Yat-Sen University, Guangzhou, 510080 Guangdong China

**Keywords:** Autism spectrum disorder, Whole-exome sequencing, De novo mutations, Pathways, Intellectual disability, Intelligence quotient

## Abstract

**Background:**

Autism spectrum disorder (ASD) is often accompanied by intellectual disability (ID). Despite extensive studies, however, the genetic basis for this comorbidity is still not clear. In this study, we tried to develop an analyzing pipeline for de novo mutations and possible pathways related to ID phenotype in ASD. Whole-exome sequencing (WES) was performed to screen de novo mutations and candidate genes in 79 ASD children together with their parents (trios). The de novo altering genes and relative pathways which were associated with ID phenotype were analyzed. The connection nodes (genes) of above pathways were selected, and the diagnostic value of these selected genes for ID phenotype in the study population was also evaluated.

**Results:**

We identified 89 de novo mutant genes, of which 34 genes were previously reported to be associated with ASD, including double hits in the EGF repeats of *NOTCH1* gene (p.V999M and p.S1027L). Interestingly, of these 34 genes, 22 may directly affect intelligence quotient (IQ). Further analyses revealed that these IQ-related genes were enriched in protein synthesis, energy metabolism, and amino acid metabolism, and at least 9 genes (*CACNA1A, ALG9, PALM2, MGAT4A, PCK2, PLEKHA1, PSME3*, *ADI1*, and *TLE3*) were involved in all these three pathways. Seven patients who harbored these gene mutations showed a high prevalence of a low IQ score (< 70), a non-verbal language, and an early diagnostic age (< 4 years). Furthermore, our panel of these 9 genes reached a 10.2% diagnostic rate (5/49) in early diagnostic patients with a low IQ score and also reached a 10% diagnostic yield in those with both a low IQ score and non-verbal language (4/40).

**Conclusion:**

We found some new genetic disposition for ASD accompanied with intellectual disability in this study. Our results may be helpful for etiologic research and early diagnoses of intellectual disability in ASD. Larger population studies and further mechanism studies are warranted.

**Supplementary Information:**

The online version contains supplementary material available at 10.1186/s40246-022-00427-7.

## Background

Autism spectrum disorder (ASD, [DSM-5]) is a group of neuronal developmental disorders that are characterized by defects in social interaction and verbal communication, together with restricted and repetitive behaviors. Other than these core symptoms, ASD may be companied by many other problems, such as intellectual disability (ID) [1], deficits in fine motor skills, speech language delay [2], metabolic disturbance of amino acids [3] or fatty acid [4], and epilepsy [5]. In addition, gastrointestinal problems, epilepsy, and sleep disorders are common phenotypes in ASD [6].

Twin and family studies revealed that genetic factors compose a major contributor for ASD. Those genetic effects can be acquired via a “new” mutation occurring in probands (de novo mutation) or harmful variants transmitted from parents. By using large-scale genome sequencing, various de novo variants have been identified in a number of genes that may be associated with the pathogenesis of ASD. For example, de novo mutations affecting GABAergic neuronal circuits [7], cytoskeletal organization, ion transport [8], ubiquitination pathway, protein synthesis and degradation, the development, formation, and function of synapses [6], and the balance in excitation and inhibition synaptic input [9], have been reported to be associated with the occurrence of ASD, demonstrating the role of de novo variants in the etiology of ASD. More interestingly, some de novo altering genes were also indicative of other clinical entities [9]. For instance, genes located on the X chromosome have been reported to contribute to ASD subgroups with ID [10], while other ASD genes are thought to be related to speech-impairment [11]. Some researchers also found an etiological overlap between ASD and epilepsy [12]. Additionally, an ASD-associated de novo mutation found in dopamine transporter (DAT T356M) can alter striatal dopamine neurotransmission and cause dopamine-dependent behaviors in mice, which is also seen in attention-deficit/hyperactivity disorder (ADHD) [13]. Therefore, tests of de novo mutation are thought to be contributable significantly to ASD research and diagnosis [14–16]. However, the genetic basis of these comorbidities in ASD remains largely unknown. Linking genetic factors to a certain symptom or particular sets of ASD may be more useful for etiologic research and potentially for diagnosis purpose. Among these comorbidities, ID is particularly relevant due of its high prevalence, high degree of heritability [10], and long-term effects on quality of life, even after entering adulthood [1].

We therefore implemented whole-exome sequencing (WES) of ASD samples in an attempt to establish a genetic architecture of ASD patients who are accompanied by certain clinical entities such as ID. To this end, we developed an analyzing pipeline to search for de novo mutation and pathways that could be related to ID phenotype in ASD.

## Results

### Clinical characteristics of subjects

In total, 79 ASD families including siblings without ASD and both healthy parents were collected in this study. Among these, 77 families were trios, while other two were quarters. As for probands, there were 72 boys and 7 girls, with mean age of 3.18 ± 1.24 years. Clinical information of patients was collected during diagnostic and follow-up visits. A considerable proportion of patients were found to have low developmental quotient (DQ) or intelligence quotient (IQ) (< 60, ~ 46.5%), non-verbal language (~ 61.0%), walking age equal with or later than 12 months (~ 84.9%), metabolic disturbance in plasma of short-median-chain acylcarnitines (~ 73.3%), thyroid hormones (~ 24.7%), and long-chain acylcarnitines (~ 17.3%); and as for plasma amino acid, the prevalence of aberrant hydroxyproline was common (~ 41.3%). The abovementioned phenotypes were further analyzed. We found that patients who were diagnosed at < 3 years of age (Fisher’s exact test, *P* = 6.15 × 10^–7^, odds ratio [OR] = 13.96), or DQ/IQ < 60 (Fisher’s exact test, *P* = 0.038, OR = 2.77) tended to have worse language ability, but no significant association was observed between diagnosed age and walking age (Fisher’s exact test, *P* = 0.12, OR = 2.81).

### *Identification of *de novo* mutations*

All subjects in this study were tested by whole-exome sequencing. On average, we produced 16.2 GB of raw reads for each sample, and 96.6% of them were mapped to the human reference genome (hg19 version) by Burrows–Wheeler Aligner (BWA). The coverage of the targeted sequences per sample ranged from 98 × to 171 × (average 119 ×), and the coverage of targeted sequences that covered at least 10 times of each sample ranged from 92.9 to 95.8% (average 94.8%, Additional file 1: Table S1). All the data showed that the sequencing data quality was relatively good for de novo mutation detection. Moreover, no exceedance of Mendelian errors was found in our data (Additional file 1: Figure S1), and all these 79 ASD families had identification of de novo mutations performed.

After validated by Sanger sequencing, we confirmed 82 de novo coding single nucleotide variants (SNVs) and 7 de novo coding insertions and deletions (INDELs) (Additional file 2: Table S2). Among these mutations, one missense and one stop-loss mutation occurred in unaffected siblings (the last two mutations in Additional file 2: Table S2). Considering the limited mutation number in siblings, totally 87 de novo events, including 57 missense mutations, 19 silent SNVs, and 4 stop gains, and 7 INDELs in probands were further analyzed. None of these abovementioned mutations were found in our in-house exome sequencing database including 2000 Han Chinese.

Additionally, we performed splicing site prediction to detect potential splice sites (detailed in ***Methods***), 3 silent and 4 missense mutations passed our threshold, and were marked as silent-splicing and missense-splicing, separately. Meanwhile, we analyzed inherited SNVs and INDELs possibly related to ASD and found 39 homozygous mutations, 3025 compound heterozygous mutations, and X-linked mutations (data not shown). We have analyzed these inherited mutations; however, no common characteristics in pathways between the inherited and de novo mutations were found in this study. Therefore, these inherited mutations will be analyzed in reports to follow.

There were about 65% of children (51/79) carrying at least one de novo SNV or INDEL. The number of each family (1.01 for each individual, on average) followed a Poisson distribution (Additional file 1: Figure S2), which suggested that there was no obvious system bias in the process of sequencing and de novo mutation detection. The average number and rate of de novo SNV/INDEL were 1.01 /0.089 and 1.51 × 10^–8^/1.32 × 10^–9^, respectively (Additional file 1: Table S3).

Compared with general mutations, the de novo mutations found in ASD children are more inclined to have a prominently higher ratio between non-synonymous (including missense, stop gain, canonical and predicted splicing sites) and synonymous de novo SNVs (NS:S = 4.0), which exceeds the expected value under a random model (NS:S = 2.85 × 10^–3^) [9], and private inherited mutations (NS:S = 1.87, *P* = 2.89 × 10^–3^) (Additional file 1: Table S4). Simultaneously, the rate of LoF mutations (loss of function mutations, including stop gain, canonical and predicted splicing sites, and frameshift INEDL, which result in the gene product having less or no function) of de novo mutations found in our data are observed to be much higher than that of private inherited mutations (*P* = 1.60 × 10^–7^), and that of de novo mutations found in the reported control [9] (*P* = 2.89 × 10^–3^) (Additional file 1: Table S4).

**Fig. 1:**
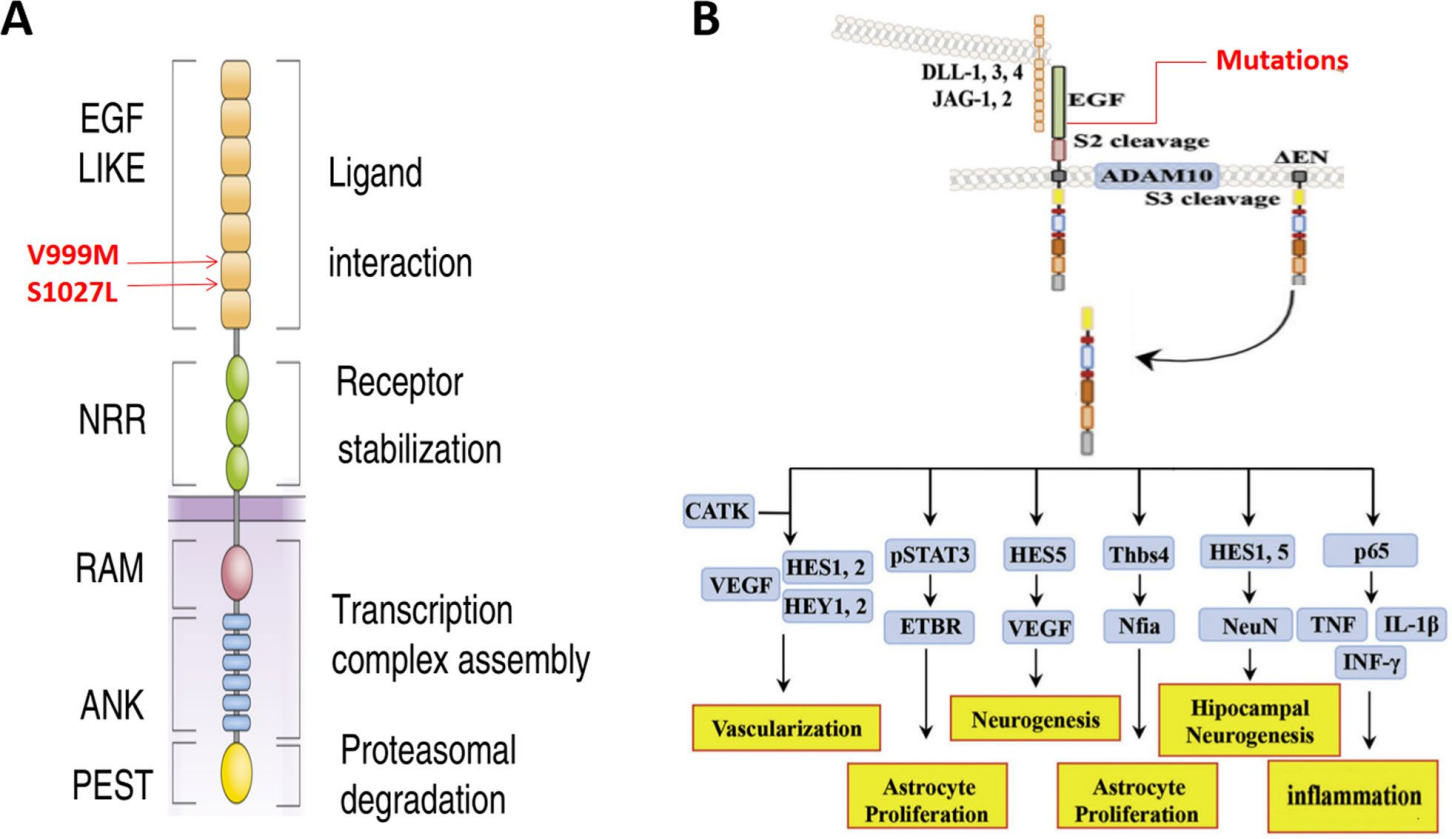
Schematic diagram and potential roles of de novo mutations in *NOTCH1* genes in neurodevelopmental diseases. **A**. Schematic diagram of *NOTCH1* gene, and de novo mutations (p.V999M and p.S1027L) found in this study. **B**. The potential roles of NOTCH1 signaling in neurodevelopmental diseases. This figure was adapted from Sanchez-Martin and Ferrando [24] and Arumugam et al. [25]

Consistent with previous ASD studies [17], we found *NOTCH1* gene recurrently mutated in 2 families, p.V999M and p.S1027L located in EGF repeats (EGF_CA domain, cd00054, Fig. 1). This gene plays an important role in NOTCH signaling pathway and is essential for neural development [18]. Besides, five other genes known to be associated with the occurrence of ASD (*CACNA1A* [19], *CHRM3* [20], *CNOT3* [21], *EPHA6* [22], and *CDH2* [23]) were detected. We also found 16 genes associated with ASD, as shown in Table 1. Because the etiology of ASD and ID overlaps genetically [10], these abovementioned 22 genes might directly affect patients’ DQ/IQ.

**Table 1:** Genes with de novo harmful mutations in this study and reported database

Gene	This study		ASD virulence gene				ASD-related gene				Mental disease-related gene				Developmental disease-related gene				Combined*	References
	Lof	Mis	Lof	Mis	CNV	N/Y	Lof	Mis	CNV	N/Y	Lof	Mis	CNV	N/Y	Lof	Mis	CNV	N/Y	Lof/Mis/CNV	(ALL diseases)
*Recurrent in this study*																				
*NOTCH1*	0	2	0	0	0	Y	0	0	0	Y	0	0	0	N	0	0	0	N	2	[17]
*Recurrent in reported database*																				
*CACNA1A*	1	0	1	0	0	Y	0	1	0	Y	0	0	0	Y	0	2	0	Y	4	[26]; [27]
*CHRM3*	0	1	0	0	0	Y	0	0	0	Y	0	0	1	Y	0	0	0	Y	1	[20]; [28]
*CNOT3*	0	1	0	0	0	Y	0	0	0	Y	0	0	0	N	0	0	0	Y	1	[21]; [29]; [30]
*EPHA6*	0	1	0	0	0	Y	0	0	0	Y	0	0	0	N	0	0	0	Y	1	[22]
*CDH2*	0	1	0	0	0	Y	0	0	0	Y	0	0	0	N	0	0	0	N	1	[23]; [31]
*KIF5C*	0	1	0	0	0	N	0	1	0	Y	0	0	0	N	0	1	0	Y	3	[32]
*KIF1A*	0	1	0	0	0	N	0	1	0	Y	0	0	0	N	0	0	0	N	2	[33]
*IKZF4*	0	1	0	0	0	N	0	0	1	Y	0	0	0	N	0	0	0	Y	2	[34]
*SEC31B*	0	1	0	0	0	N	0	0	0	Y	0	0	0	N	0	0	0	N	1	[35]
*LMO7*	1	0	0	0	0	N	0	0	0	Y	0	0	0	N	0	0	0	N	1	[36]
*MYCBP2*	0	1	0	0	0	N	0	0	0	Y	0	0	0	N	0	0	0	N	1	[37]
*PALM2*	1	0	0	0	0	N	0	0	0	Y	0	0	0	N	0	0	0	N	1	[38]
*ALG9*	0	1	0	0	0	N	0	0	0	Y	0	0	0	N	0	0	0	N	1	[39]
*WDTC1*	0	1	0	0	0	N	0	0	0	Y	0	0	0	N	0	0	0	N	1	[40]
*UBR4*	0	1	0	0	0	N	0	0	0	Y	0	0	0	Y	0	0	0	N	1	[41]; [42]
*MKL1*	0	1	0	0	0	N	0	0	0	Y	0	0	0	N	0	0	0	N	1	[43]
*CREB5*	0	1	0	0	0	N	0	0	0	Y	0	0	0	N	0	0	0	N	1	[44]
*AOX1*	0	1	0	0	0	N	0	0	0	Y	0	0	0	N	0	0	0	N	1	[45]
*TLE3*	1	0	0	0	0	N	0	0	0	Y	0	0	0	N	0	0	0	N	1	[46]
*ARID5B*	1	0	0	0	0	N	0	0	0	Y	0	0	0	N	0	0	0	N	1	[47]
*PHACTR3*	0	1	0	0	0	N	0	0	0	Y	0	0	0	N	0	0	0	N	1	dbGaP^$^: phs000267.v5.p2 (NIMH Autism Genome Project)
*HOMER2*	0	1	0	0	0	N	0	0	0	N	0	0	0	Y	0	0	0	Y	1	[48]; OMIM616707
*XPNPEP1*	1	0	0	0	0	N	0	0	0	N	0	0	0	Y	0	0	0	N	1	[49]
*PTPRM*	0	1	0	0	0	N	0	0	0	N	0	0	1	Y	0	0	0	N	2	[50]
*MFAP1*	0	1	0	0	0	N	0	0	0	N	0	0	1	Y	0	0	0	N	2	[51]
*SEC31A*	0	1	0	0	0	N	0	0	0	N	0	1	0	Y	0	0	0	N	2	[52]
*MASP1*	0	1	0	0	0	N	0	0	0	N	0	0	0	Y	0	0	0	Y	1	[53]
*CFH*	0	1	0	0	0	N	0	0	0	N	0	0	0	Y	0	0	0	N	1	[54]
*YWHAQ*	0	1	0	0	0	N	0	0	0	N	0	0	0	Y	0	0	0	N	1	[55]
*MGAT4A*	1	0	0	0	0	N	0	0	0	N	0	0	0	Y	0	0	0	N	1	[56]
*TMEM8B*	0	1	0	0	0	N	0	0	0	N	0	0	0	Y	0	0	0	N	1	[57]
*ABCA5*	0	1	0	0	0	N	0	0	0	N	0	0	0	Y	0	0	0	N	1	[58]
*LYST*	0	1	0	0	0	N	0	0	0	N	0	0	0	Y	0	0	0	Y	1	[59]; OMIM214500
*POU2F2*	0	1	0	0	0	N	0	0	0	N	0	0	0	Y	0	0	0	N	1	[60]
*NOL6*	0	1	0	0	0	N	0	0	0	N	0	0	0	Y	0	0	0	N	1	MCID#: ATS383 MIFTS: 37

All mutations in this table mean de novo mutations found in cases

*Combined number of mutations including this study and the references cited

^#^MalaCards database: https://www.malacards.org/

^$^The database of Genotypes and Phenotypes (dbGaP): https://www.ncbi.nlm.nih.gov/gap/

OMIM: Online Mendelian Inheritance in Man https://www.ncbi.nlm.nih.gov/omim

Additionally, there were other eight de novo altering genes reported in mental diseases (*KCNJ13*, *H2AFX*, *ZYX*, *MAST2*, *MARK2*, *ADI1*, *PLEKHA1*, and *PCK2*) [61–64], and four ones associated with developmental diseases (*PDE3B*, *PIEZO1*, *HEYL*, and *CELSR1*) [65–67].

We then further compared de novo mutations in diverse sub-population based on the clinical information, such as diagnosed early (< 3 years), walking later (> 12 months), DQ/IQ (< 60), language impairment, and abnormal plasma levels of short-median-chain/long-chain acylcarnitine, hydroxyproline, and thyroid function shown as Additional file 1: Table S5. Compared with other patients, LoF mutations were more likely to occur in those with abnormal plasma thyroid function levels (Fisher’s test, *P* = 4.27 × 10^–3^, OR = 11.10).

### De novo* disruptions of genes and pathways in subgroups in ASDs*

Eighty-six de novo altering genes were annotated by GO (http://www.geneotology.org) and KEGG pathway database (http://www.genome.jp/kegg/pathway.html) and were grouped into five combined pathways, which were related to protein synthesis, pressure, energy metabolism, development, and amino acid metabolism, respectively (Additional file 2: Table S6).

We performed association analyses between the above pathways and the ID phenotype (DQ/IQ < 60). And we found that the pathways related to protein synthesis, energy metabolism, and amino acid metabolism were significantly associated with the DQ/IQ levels (*P* values 0.019, 0.008, and 0.034, respectively) (Table 2).

**Table 2:** Association of mutations in selective pathways with clinical phenotypes

Phenotypes	Protein synthesis		Pressure		Energy metabolism		Development		AA metabolism	
	Related	Unrelated	Related	Unrelated	Related	Unrelated	Related	Unrelated	Related	Unrelated
Total genes	47	24	23	48	15	56	37	34	12	59
*IQ/DQ*										
< 60	16	17	7	26	2	31	18	15	2	31
≥ 60	22	6	11	17	10	18	15	13	8	20
*P*	0.019		0.162		0.008		1.00		0.034	
OR	0.26		0.42		0.12		1.04		0.17	

*IQ* intelligence quotient. *DQ* developmental quotient

### Protein–protein interaction networks and mutations on key networks

**Fig. 2:**
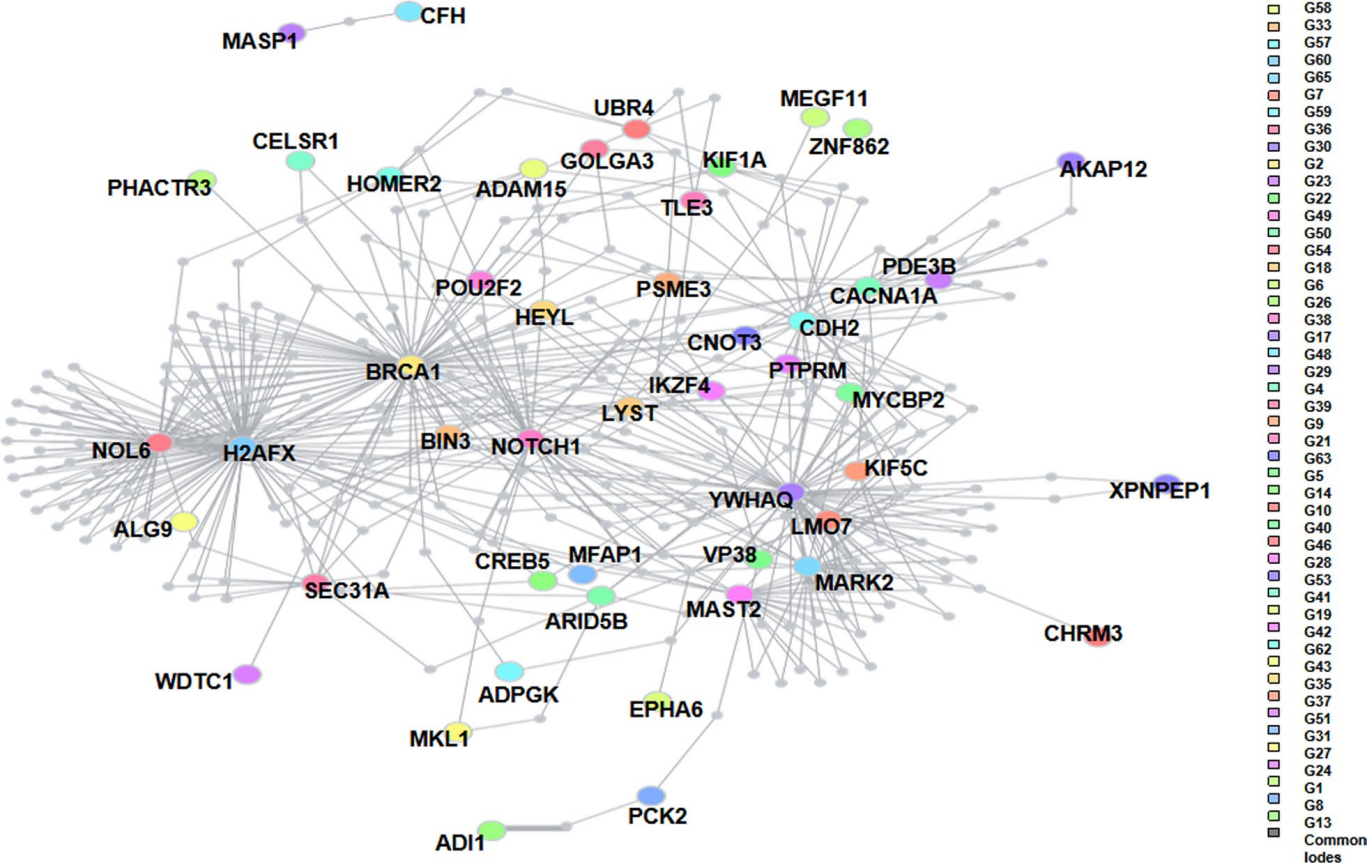
Protein–protein interaction for all de novo altering genes

For all genes with potential harmful de novo mutations, protein–protein interaction networks were predicted by DAPPLE (Disease Association Protein–Protein Link Evaluator) and STRING (http://string-db.org). There were more than 40 nodes in the protein–protein interaction networks (Fig. 2). Most of these nodes (genes) are relative to ASD, mental, or developmental diseases.

Interestingly, nine genes in these networks (*CACNA1A, ALG9, PALM2, MGAT4A, PCK2, PLEKHA1, PSME3*, *ADI1*, and *TLE3*) are concurrently involved in all above important pathways (protein synthesis, energy metabolism, and amino acid metabolism). All of these 9 genes were reported to be related to brain development. For example, 4 of them are relevant to ASD (*CACNA1A, ALG9, PALM2,* and *TLE3*). And the other 4 genes have been associated with schizophrenia (*MGAT4A* [56]), Leigh syndrome (*ADI1*, OMIM: 256,000), congenital hypomyelinating neuropathy (*PLEKHA1*, OMIM: 605,253), and ID (*PCK2* [64]), respectively. Additionally, another gene *PSME3* is also involved in the brain development, which is an e-QTR loci for the expression on hippocampus, basal ganglia, frontal cortex, cerebellum, and anterior cingulate cortex, and so on [56].

Moreover, cases that carried these mutations were all males and showed a high prevalence of DQ/IQ < 70 (6/7), non-verbal language (5/7), and an early diagnostic age (< 4 years, 7/7) (Table 3). Combined with the above results, it implies that the metabolism pathways of amino acid/protein/energy are relative with the etiology of intellectual disability in ASD.

**Table 3:** Clinical phenotypes in carries with the mutations in genes involving the three important pathways

Case	Gene	Sex	Diagnosed age	Father age(y)*	Mother age (y)*	DQ/IQ	Language	Walking age (m)	Hydroxyproline	Thyroid function	C13-C18	C0-C6
K2	*ALG9 PALM2*	M	2.3	33	31	68	NV	13	Normal	Normal	Normal	C2\C5 increased
AL4	*MGAT4A*	M	3	33	34	55	NV	13	NA	Normal	Normal	C2 increased
D3	*PCK2 CACNA1A*	M	2.8	–	–	NA	NV	15	Decreased	Normal	Normal	Normal
AM5	*PLEKHA1*	M	3.2	28	26	61	NV	11	Normal	Normal	Normal	C3 increased
AG7	*PSME3*	M	3.4	34	33	60	V	16	NA	FT3 increased	Normal	C6 decreased
V3A	*ADI1*	M	1.9	25	23	68	NV	15	Normal	Normal	Normal	Normal
R10	*TLE3*	M	3.3	26	23	99	V	15	Decreased	Normal	Normal	C3-5 increased

* The age when proband at birth

*NV* non-verbal. *NA* non-analyzed. *FT3* free triiodothyronine

Furthermore, our panel of these 9 altering genes reached a 10.2% diagnostic rate (5/49) in early diagnostic patients with a low DQ/IQ value and also reached a 10% diagnostic yield (4/40) in patients with both a low DQ/IQ score and a non-verbal language. Our results suggested a diagnostic value of De novo mutations within metabolism networks of amino acid/protein/energy in ASD/ID comorbidity.

### The expression patterns of major disrupted pathway genes in different brain tissues

We investigated the expression level of all de novo genes based on BrainSpan (http://www.brainspan.org/static/download.html) and BrainStars (http://brainstars.org/). The expressed genes in specific brain regions (including CB, CBC, HIP, STR, AMY, and PIT) were defined by RPKM > 5 (BrainSpan: CB, CBC, HIP, STR, AMY) or top 25% expression (BrainStars: PIT). We found that the genes involved in protein synthesis, such as *YWHAQ*, *H2AFX*, *CDH2*, and *KIF1A/KIF5C*, were highly expressed in different brain regions at all periods (Additional file 1: Figure S3). As for the genes were involved in energy metabolism, such as *PSMES*, *SEC31A*, and *WDTC1*, their expressions were not significantly varied in different brain regions at various periods (Additional file 1: Figure S4). Neither the genes involved in pressure (*H2AFX*, *CDH2*, *KIF5C*, *PSMES*, and *PTPRM*, Additional file 1: Figure S5), nor those involved in amino acid metabolism (*PTPRM*, Additional file 1: Figure S6), or development (*YWHAQ*, *CDH2*, and *KIF1A/KIF5C*, Additional file 1: Figure S7). Totally, the highly expressing genes in brain were constantly expressed across different brain regions during various development periods (*YWHAQ*, *CDH2*, and *KIF1A/KIF5C*, Additional file 1: Figure S8). It implied that many periods during brain development are pivotal for the etiology for ASD.

## Discussion

In this study, we explored the genotype–phenotype relationships in ASD, to facilitate ongoing efforts to explain the molecular mechanisms of their endo-phenotypes. We found that pathways related to protein synthesis, energy metabolism, and amino acid metabolism were significantly associated with DQ/IQ levels in ASD. Those that carried the mutations in their connection node (*CACNA1A, ALG9, PALM2, MGAT4A, PCK2, PLEKHA1, PSME3*, *ADI1*, and *TLE3*) obviously exhibited low DQ/IQ and language impairment.

In this study, we found that de novo mutations in probands occurred in 86 genes, including 22 related to ASD, and 26 associated with mental/developmental diseases. Because genes related to mental and developmental diseases are also potentially associated with ASD [68, 69], the 56% de novo altering genes (48/86) in this study are likely biologically related to the occurrence of ASD. Moreover, the average number and rate of de novo SNV/INDEL, and the ratio of non-synonymous to synonymous de novo SNVs (NS:S) was similar to previous ASD studies [9]. Additionally, the quality of whole-exome sequencing is high (Additional file 1: Figures S1 and S2). Thus, we believe that our findings regarding genetic associations in ASD and ID are creditable.

To our knowledge, this is the first time that de novo mutations associated with amino acid/protein/energy metabolism have been found to play a pivotal role in the etiology of ID in ASD. In this study, the nine de novo altering genes (*CACNA1A, ALG9, PALM2, MGAT4A, PCK2, PLEKHA1, PSME3*, *ADI1*, and *TLE3*) were involved in all the above important pathways simultaneously. Interestingly, they are all reported related to brain development. Among of them, *CACNA1A* is involved in protein synthesis (GO:0,043,231 and GO:0,043,234), energy metabolism (GO:0,044,262), and amino acid metabolism (ko04010), and is reported to the occurrence of ASD [26, 27]. And *PALM2*, ALG9, and *TLE3* also participate in the above pathways such as GO:0,043,231, GO:0,006,487, ko01100, and ko04010, and all of them are reported to be ASD-relative [38, 39, 46]. *MGAT4A*, a schizophrenia-relevant gene [56], is involved in the GO:0,006,487, GO:0,043,234, and ko01100 pathways. The *ADI1* and *PLEKHA1* are involved in amino acid and derivative metabolism (R-HSA-71291), synthesis of PIPs at the plasma membrane (R-HSA-1660499) and energy metabolism (R-HSA-1430728), and are related to neurometabolic disease (Leigh syndrome; OMIM256000) and neuron developmental disease (congenital hypomyelinating neuropathy; OMIM605253), respectively. And *PCK2* is reported to be associated with the etiology of ID [64]. Meanwhile, its mutations are the cause of an inherited metabolic disease (PEPCK deficiency, mitochondrial, OMIM: 261,650), and it is also related to GO:0,043,231, ko00010, ko00620, and ko00020 pathways. Another gene *PSME3* (also named as PA28γ or REGγ) is not reported relative to ASD or mental diseases in human previously. However, it is an e-QTR loci for the expression on many ASD-relative tissues, such as hippocampus, frontal cortex, and cingulate cortex [56]. And *Psme3* gene transfer improves motor coordination in mouse model of Huntington's disease [70]. This gene is involved in the pathways of amino acid and derivative metabolism (R-HSA-71291), ABC-family proteins-mediated transport (R-HSA-382556), APC/C-mediated degradation of cell cycle proteins (R-HSA-174143), and is reported to regulate energy homeostasis [71]. Our data suggest that a novel type of targets involving nodes of the important pathways modulating protein synthesis, energy production, and neurotransmission (BCAAs) simultaneously, might better explain some severe problems in ASD, such as comorbidity with ID and language impairment.

Brain dysfunctions related to IQ and language development in ASD disrupt the transducing experience-mediated neural activity into long-term modifications of synapses [72]. In many cases, the long-term synaptic modifications rely upon new protein synthesis, including the following process: protein synthesis activated by the stimuli of neuron receptors (NMDA), then regulation of the synthesis of synaptic signaling molecules (CaMKIIα), ion channels (SK channel), translation factors (eIF4E), and glutamate receptor subunits (GluA1, GulA2) [73–75]. These mechanisms for synaptic modifications and plasticity link brain protein synthesis with ID and language learning in ASD [76]. Amino acids, especially branched chain amino acids (BCAAs) which comprise as much as 30% of proteins in the cell, are also related to long-term modifications of synapses. By studying the mutation of *BCKDK* (a metabolizing enzyme of BCAAs) [77, 78] and *SLC7A5* (a neutral amino acid transporter) [79], people know that these amino acids are also used as neurotransmitters and as metabolic intermediates in the etiology of ASD, ID, and other mental diseases [80]. Moreover, oxygen consumption, a major index for energy metabolism in the brain, accounts for about one-fifth of the total consumption of the human body. It has been proven that glycolysis and β-oxidation of fatty acid are important mechanisms closely related to brain development dysfunction in ASD [4]. Therefore, it makes sense that energy metabolism-related pathways play a vital role in the etiology of ID and language impairment in ASD. Taken together, our findings on the genetic association between the networks of amino acid/protein/energy-metabolism and ID in autism are biological feasible.

Gene panel sequencing is thought to be helpful for screening ID phenotype in ASD patients. For instance, Redin et al. [81] reached a 25% diagnostic yield of ID/ASD comorbidity in 106 selected patients without congenital malformations, fragile X syndrome, or detectable CNV mutations, using a panel with 99 X-linked and 118 autosomal genes. Grozeva et al. [82] reported an 11% diagnostic rate on unselected 986 ASD patients with moderate to severe ID, using a larger panel of 565 genes. Aspromonte et al. [83] designed a smaller panel including 74 genes related to both ID and ASD, and reached a 27% diagnostic rate (41/150) in a careful selected ASD population with ID, who were negative for CNV and deletions/imprinting defects. By reviewing some references, Chiurazzi et al. suggested a panel of 174 genes (64 X-linked and 110 autosomal) to screen ID/ASD patients [10]. In this study, we suggested a panel of 9 genes to identify ASD patients with ID and non-verbal language with a 10% diagnostic yield, and it reached a similar diagnostic rate in early diagnostic ASD patients with ID. Our findings are helpful for future disease diagnosis.

Additionally, *NOTCH1* was found to have mutated recurrently in this study (c.G2995A:p.V999M and c.C3080T:p.S1027L). Human *NOTCH1* gene (Gene ID:4851) encodes a member of the NOTCH protein family (belonging to Type I transmembrane protein family), which shares a characteristic structure: multiple extracellular epidermal growth factor-like (EGF) repeats. As a receptor, extracellular EGF repeats of NOTCH1 are pivotal for binding to its ligands, such as JAG1/2 and DLL1 ~ 3 (Fig. 1A) [24, 25]. After activation of these ligands, NOTCH1 receptor is hydrolyzed by ADAM10 metalloprotease and γ-secretase complex, then releases an intracellular fragment to nuclear, and participates in transcriptional regulation of many developmental genes, thus playing important roles in neurogenesis, vascularization, inflammation, and other processes (Fig. 1B) [17, 18, 25]. In this study, both V999M and S1027L de novo mutations were located in the EGF repeats, influencing the binding of NOTCH1 receptor with its ligands, disconnecting the networks of neuron-neuron and/or neuron/stroma cell, and hindering brain development, thus leading to the occurrence of ASD. Therefore, we postulate that the recurrent mutations in EGF_CA domain of *NOTCH1* are related to and may be a risk factor of ASD. We believe these findings would be valuable for future etiological study.

This study have some limitations: Our results must be interpreted with caution given the small sample sizes of both studies and challenges inherent in combining datasets.

## Conclusion

Our data suggest that the connection nodes of the pathways such as amino acid/protein/energy-metabolism should be a novel type of target for ASD, which may play a vital role in the etiology of ID in ASD. Our findings suggest a panel of 9 genes to screen ASD patients with ID and language delay in this study. Moreover, the recurrent mutations in EGF_CA domain (EGF repeats) of *NOTCH1* are associated with ASD, which implies a new disease mechanism. However, studies with larger population in different ethnic groups and functional studies are warranted to validate our findings.

## Methods

### Study population

From Oct 2015 to Jan 2017, we collected 79 children with ASD from a National Women and Children’s Medical Center for the south central region in China. All these 79 patients (77 trios and 2 quarters) (male/female = 72/7; 3.19 ± 1.24 years) met the following inclusion criteria consisting of Diagnosis and statistical Manual of Mental Diseases version-5 (DSM-5), Autism Diagnostic Interview-Revised (ADI-R), and Autism Diagnostic Observation Schedule (ADOS), and those who were initially diagnosed at the age of fewer than two years old would be followed up to obtain the definitive diagnosis when whose age was at least two years old. All included subjects had an extensive clinical evaluations including relevant demographic data collection, neurological assessments, developmental quotient (DQ) assessment by Gesell Development Diagnosis Scale (GDDS)/ intelligence quotient (IQ) assessment by Chinese Wechsler Intelligence Scale for children- IV Version (CWISC-IV) or by Chinese Wechsler Young Children Scale of Intelligence-IV Version (CWYCSI-IV), and the testing of plasma levels of amino acids, acylcarnitines (C0-C18) via HPLC-GC/MS as well as thyroid function. Metabolic disturbances in plasma hydroxyproline, acylcarnitines, and thyroid function were defined as theirs levels increased or decreased more than twofold as compared to the norm reference. The non-verbal autistic child was defined as a child with spontaneous functional words less than five clinically. The study was approved by the Clinical Research Ethics Committee of Guangzhou Women’s and Children's Medical Center, and informed consent for participation was obtained from either of their parents/guardians. Blood samples of the probands, parents, and other available relatives including siblings were obtained from who gave informed consent.

### Exome capture and sequencing

Genomic DNA of the studied families (proband, both parents, and other available siblings) was extracted from the peripheral blood using the QIAamp DNA Blood Mini kit (QIAGEN GmbH, Hilden, Germany). We quantified initial DNA using a Qubit High Sensitivity Assay and checked sample purity using the Nanodrop OD260/280 ratio. Purified DNA was fragmented into an average size of 250 bp and hybridized by the Agilent V5 sequence capture array to capture the exonic DNA. We performed whole-exome sequencing with 100 bp pair-end reads on Illumina HiSeq 4000 platform following Illumina’s recommended protocol. The raw image files were processed using the standard Illumina Pipeline (version 1.3.4) for base calling with the default parameters.

### Alignment and variant calling

After removing reads caught adapter sequence and low-quality sequences (rate of base with quality < 5), the sequencing quality of all processed FASTQ files was measured by Fastqc (version 0.11.4). Pruned reads in the FASTQ format were aligned to the human reference genome (hg 19 version) by BWA (version 0.5.9-r16), and the duplicated sequence generated in the processing of PCR was marked by Picard (http://broadinstitute.github.io/picard). We utilized the Genome Analysis Toolkit (GATK; version 3.5) to perform the local realignment and base quality recalibration in the sequencing target region and its extension (500 bp) region and thereby obtained an ‘Analysis-Ready’ BAM file for each individual. The single nucleotide variants (SNV) and insertions and deletions (INDELs) were jointly called by HaplotypeCaller in GATK for every three or four members per family, and FamSeq was used to adjust variants based on family information. We further removed the mutations with a Variant Quality Score logs odds ratio (VQSLOD) with a tranche sensitivity of less than 99.9% to alleviate other confounders’ effects. All output files, which generated in the universal variant call format (VCF), were annotated by ANNOVAR with various databases.

### Sample quality control

Two methods were adopted for quality control checks in all samples: (1) Genotypes of 24 common mutations (frequency > 0.4 in Eastern Asian of 1000 Genome Project) were tested by Mass Spectrum, and the concordance of initial DNA’s genotype and sequencing data should be no less than 0.95; (2) Mendelian rate of each family should be no larger than 0.5%.

### Splicing site prediction

We used three tools (including NetGene2, SplicePort, and Human Splicing Finder) to predict whether a silent or missense de novo mutation can lead to candidate transcript splicing. Mutations judged as candidate splicing sites by at least two of above-mentioned programs would be marked as silent-splicing or missense-splicing and would be regarded as splicing mutation in this study.

### Inherited mutations

In addition to de novo mutations, three types of inherited mutations that may lead to ASD were also extracted: (1) rare (minor allele frequency < 1% in East Asian of 1000 Genome Project and ExAC) homozygous coding mutations that transmitted from heterozygous parents; (2) rare compound heterozygous coding mutations that transmitted from heterozygous parents; (3) rare heterozygous coding mutations of male proband, which transmitted from maternal X chromosome. We also picked up the private inherited mutations (rare heterozygous mutations that inherited from father or mother, and only observed in single family) to compare with the de novo mutations. All above-inherited SNVs and INDELs have a good genotype quality (phred values greater than 20, sequencing depth larger than 10 ×).

### Pathways, protein–protein interaction, and co-expression networks

All de novo altering genes were annotated by GO (http://www.geneotology.org) and KEGG pathway database (http://www.genome.jp/kegg/pathway.html). The protein–protein interaction networks of these altering genes were constructed for all potential harmful genes based on DAPPLE (Disease Association Protein–Protein Link Evaluator) and STRING (http://string-db.org), and the co-expression network was built with the BrainSpan Atlas resource. Expression data of samples before early childhood (age < 6 years) in multiple brain regions (including CBC, CB, HIP, AMY, and STR) were used. We used person test to estimate the co-expression based on periods and brain regions, respectively.

### Statistical analysis

Chi-square test and logistic analysis were used to analyze the data in standard R packages. A two-sided *P* value of < 0.05 defined statistical significance.

## Supplementary Information

**Additional file 1.** **Figure S1:** Mendelian error rate of all families. **Figure S2:** The number of de novo mutations in probands and siblings followed Poisson distribution. **Figure S3:** Gene (protein synthesis) expression in different brain regions at various periods. **Figure S4:** Gene (energy) expression in different brain regions at various periods. **Figure S5:** Gene (pressure) expression in different brain regions at various periods. **Figure S6:** Gene (amino acid) expression in different brain regions at various periods. **Figure S7:** Gene (development) expression in different brain regions at various periods. **Figure S8:** Gene expression in different brain regions at various periods. **Table S1:** The general sequencing information of all ASD families. **Table S3:** De novo SNV/InDel rate in all ASD families. **Table S4:** The de novo mutations and private inherited mutations of ASD in our study, and de novo mutations in reported studies, and unaffected control in reported studies. **Table S5:** Comparison of de novo mutations in diverse sub-population based on clinical information.

**Additional file 2.** **Table S2:** De novo mutations confirmed in 79 probands and their 2 siblings (Excel file) (dataset1). **Table S6:** De novo altering genes that involved in protein synthesis, pressure, energy metabolism, development, and amino acid metabolism (Excel file) (dataset2).

## Data Availability

The datasets used and/or analyzed during the current study are included in supplementary files, and the source data are available from the corresponding author on reasonable request.
