# Supplementary material for: De novo mutations within metabolism networks of amino acid/protein/energy in Chinese autistic children with intellectual disability
Source: Hum Genomics. 2022 Nov 1;16:52. doi: 10.1186/s40246-022-00427-7 (PMC9623983; doi:10.1186/s40246-022-00427-7)
Supplement: Supplementary file 1 — Additional file 1. Figure S1: Mendelian error rate of all families. Figure S2: The number of de novo mutations in probands and siblings followed Poisson distribution. Figure S3: Gene (protein synthesis) expression in different brain regions at various periods. Figure S4: Gene (energy) expression in different brain regions at various periods. Figure S5: Gene (pressure) expression in different brain regions at various periods. Figure S6: Gene (amino acid) expression in different brain regions at various periods. Figure S7: Gene (development) expression in different brain regions at various periods. Figure S8: Gene expression in different brain regions at various periods. Table S1: The general sequencing information of all ASD families. Table S3: De novo SNV/InDel rate in all ASD families. Table S4: The de novo mutations and private inherited mutations of ASD in our study, and de novo mutations in reported studies, and unaffected control in reported studies. Table S5: Comparison of de novo mutations in diverse sub-population based on clinical information. [file 40246_2022_427_MOESM1_ESM.docx]

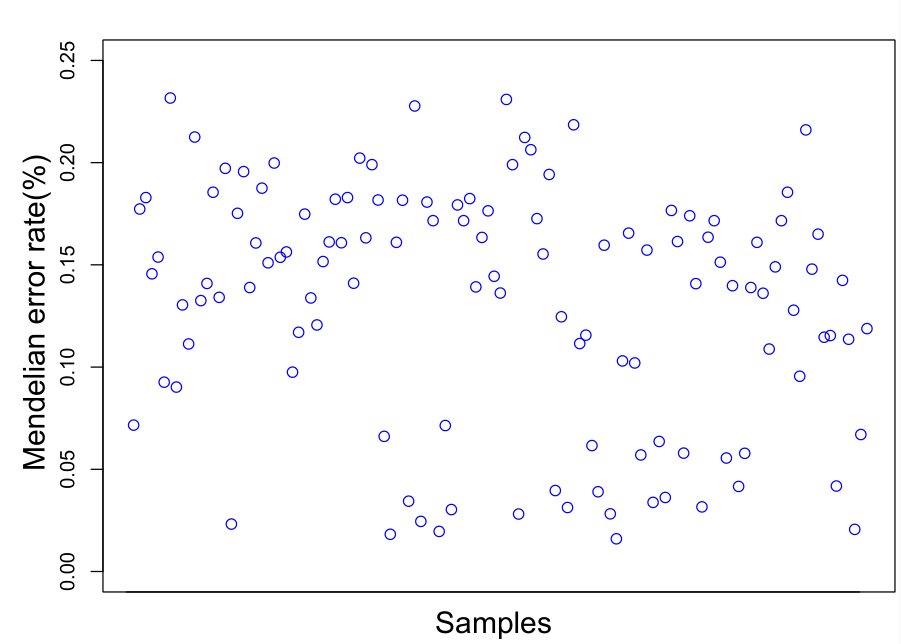


**Figure S1**


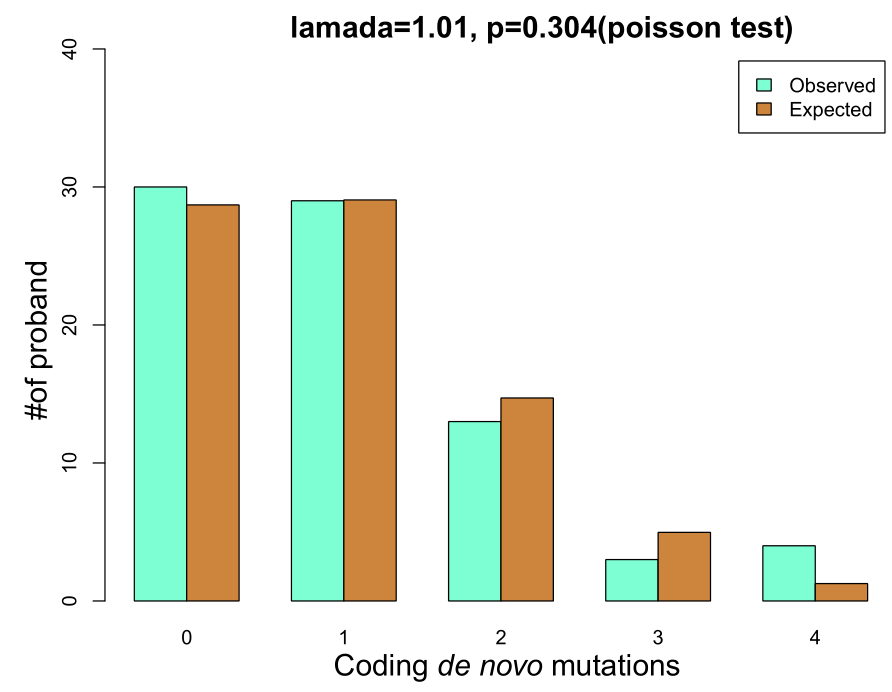


**Figure S2**


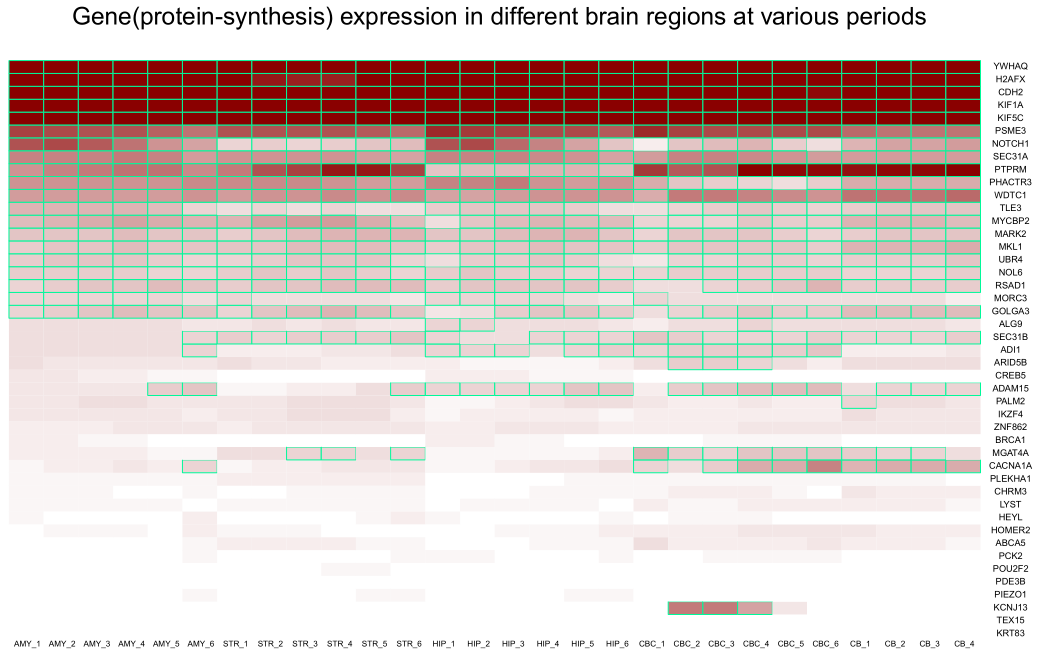


**Figure S3**


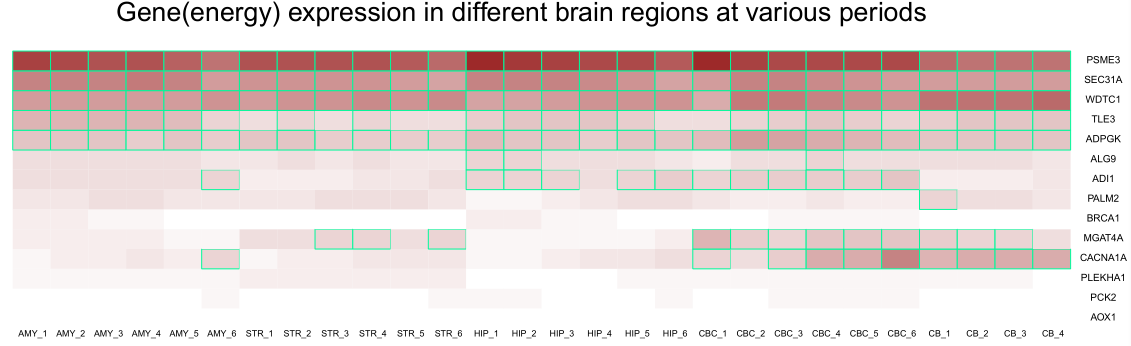


**Figure S4**


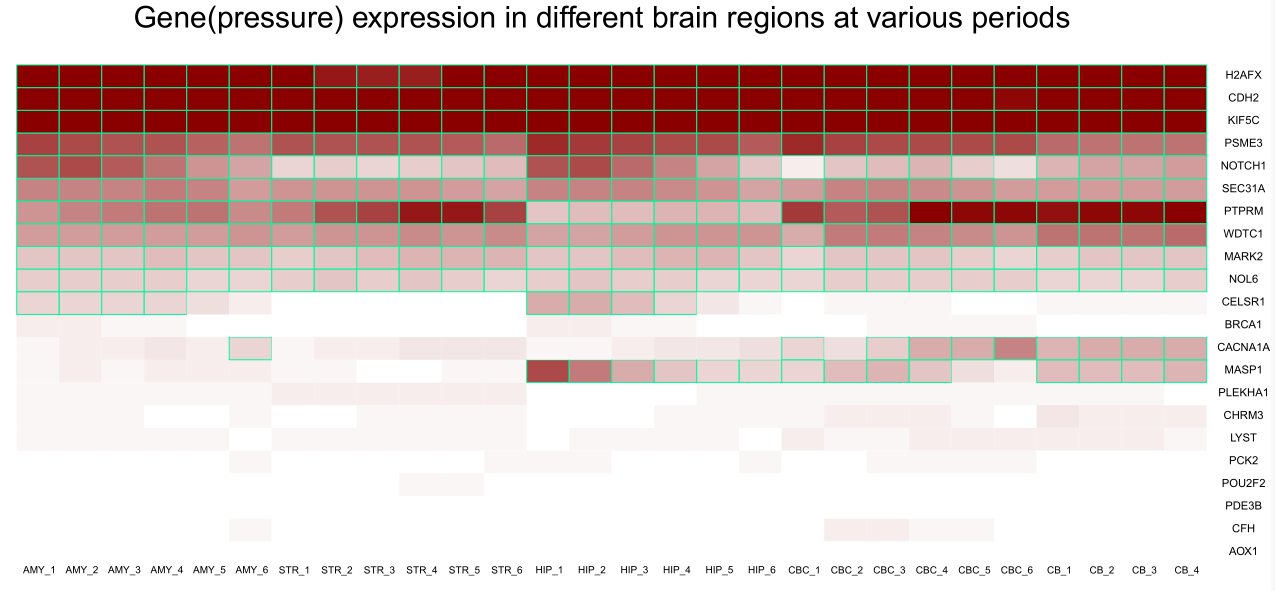


**Figure S5**


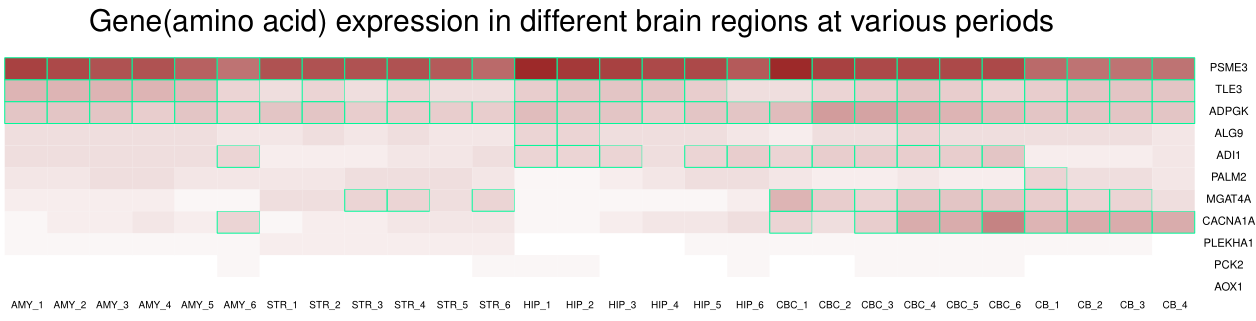


**Figure S6**


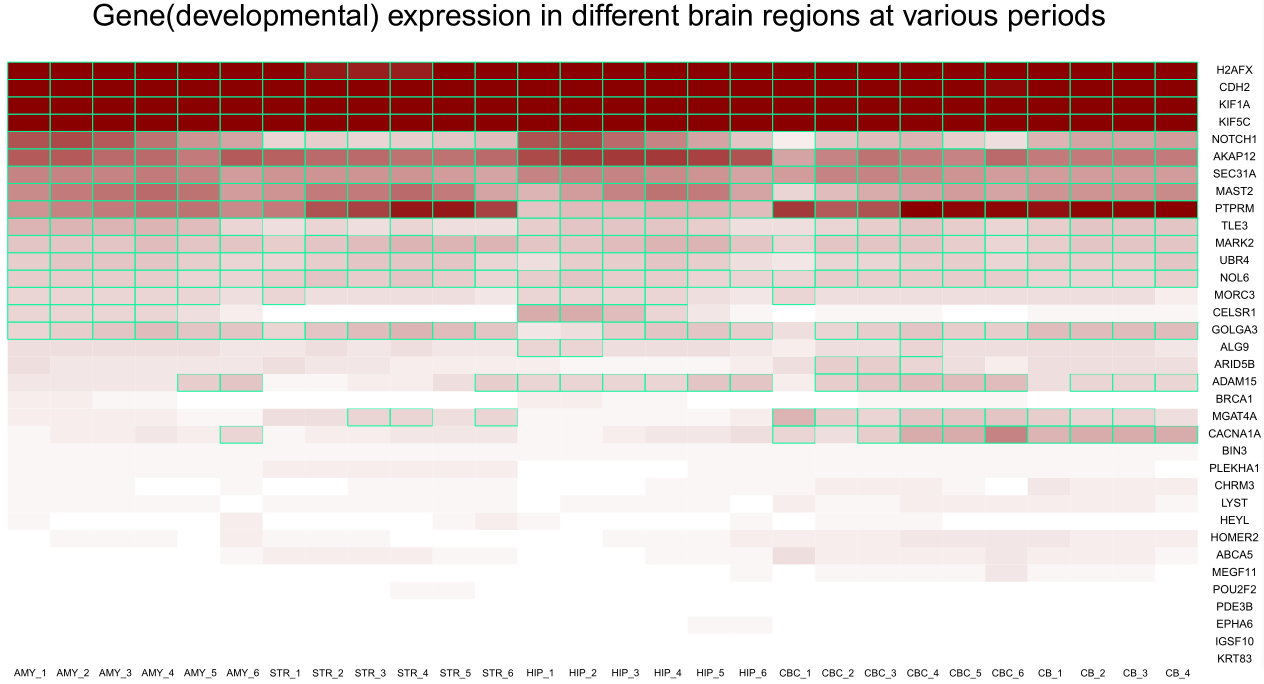


**Figure S7**


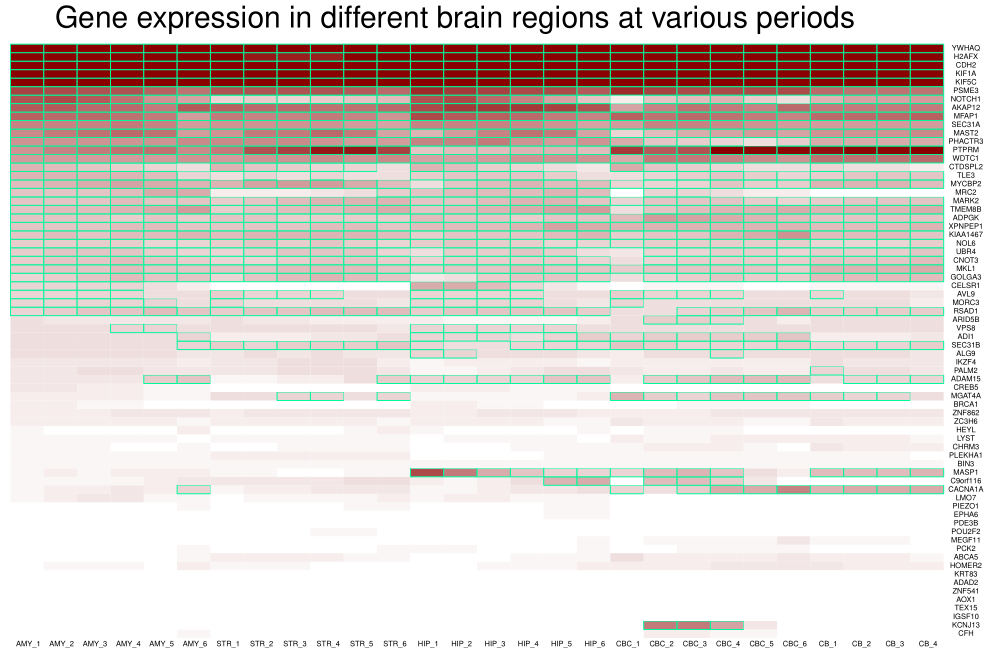


**Figure S8**

**Table S1.** The general sequencing information of all ASD families

|  | Mean ± Standard deviation |
| --- | --- |
| Read length (bp) | 100 |
| Number of individuals | 239 |
| Raw reads (Gb) | 17.29±2.13 |
| Mapped reads on target region (Gb) | 6.14±0.45 |
| Mapping rate (%) | 96.64±0.71 |
| Average sequencing depth (fold) | 119.09±8.80 |
| Proportion of target region covered ≥1X (%) | 98.28±0.16 |
| Proportion of target region covered ≥4X (%) | 96.38±0.18 |
| Proportion of target region covered ≥10X (%) | 94.78±0.22 |
| Proportion of target region covered ≥20X (%) | 92.32±0.49 |

**Table S2.** *De novo* mutations confirmed in 79 probands and their 2 siblings. (Excel file, shown in Additional File 2)

**Table S3.** Homozygous mutations, compound heterozygous mutations and X-linked mutations confirmed in 79 probands. (Excel file, shown in Additional File 3)

**Table S3.** *De novo* SNV/InDel rate in all ASD families

| Item | Amount of Samples | Amount of de novo mutation | De novo mutation (per sample/rate) | Non-synonymous* / frameshift | Non-synonymous / frameshift (per sample / rate) | Synonymous / nonframeshift | Synonymous / nonframeshift (per sample / rate) |
| --- | --- | --- | --- | --- | --- | --- | --- |
| Proband (SNV) | 79 | 80 | 1.01 / 1.51E-08 | 64 | 0.81 / 1.21E-08 | 16 | 0.20 / 0.30E-08 |
| Sibling (SNV) | 2 | 2 | 1.00 / 1.49E-08 | 2 | 1.00 / 1.49E-08 | 0 | 0 / 0 |
| Proband (InDel) | 79 | 7 | 0.089 / 1.32E-09 | 5 | 0.063 / 0.95E-09 | 2 | 0.025 / 0.38E-09 |
| Sibling (InDel) | 2 | 0 | 0 / 0 | 0 | 0 / 0 | 0 | 0 / 0 |

^*^Nonsynonymous mutations include nonsense, missense mutations, and canonical and predicted splicing sites

**Table S4.** The *de novo* mutations and private inherited mutations of ASD in our study, and *de novo* mutations in reported studies, and unaffected control in reported studies.

| Function | ASD | | Reported ASD | Reported control | Function | ASD | | Reported ASD | Reported control |
| --- | --- | --- | --- | --- | --- | --- | --- | --- | --- |
|  | *de novo* | private inherited |  |  |  | *de novo* | private inherited |  |  |
| nonsynonymous | 64 | 18867 | 708 | 406 | loss-of-function | 16 | 934 | 109 | 39 |
| synonymous | 16 | 10071 | 244 | 150 | missense | 53 | 18395 | 645 | 383 |
| nonsy/sy | 4.00 | 1.87 | 2.9 | 2.71 | LOF/missense | 0.30 | 0.05 | 0.17 | 0.1 |
| *P* (*de novo* vs private inherited mutations) | 2.89E-03 | | | | *P* (*de novo* vs private inherited mutations) | 1.60E-07 | | | |
| OR  (*de novo* vs private inherited mutations) | 2.14 | | | | OR (*de novo* vs private inherited mutations) | 5.94 | | | |
| *P* (patient vs. control) | 0.11 |  | 0.58 | - | *P*  (patient vs. control) | 1.69E-03 |  | 1.02E-02 | - |
| OR  (patient vs. control) | 1.48 |  | 1.07 | - | OR  (patient vs. control) | 2.96 |  | 1.66 | - |

Nonsynonymous mutations include nonsense, missense mutations, and canonical and predicted splicing sites, loss-of-function includes nonsense, canonical and predicted splicing sites and frameshift indel.

**Table S5.** Comparison of *de novo* mutations in diverse sub-population based on clinical informations.

| Variables | n(%) | de novo SNV (ratio) | De novo InDel (ratio) | Ns/sy | LOF /missense | Potential harmful^^^ |
| --- | --- | --- | --- | --- | --- | --- |
| Total | 79 | 80 (1.01) | 7 (0.09) | 64/16 | 16/53 | 71 |
| **Diagnosed age** |  |  |  |  |  |  |
| ≥3 years | 39 | 44(1.13) | 3(0.08) | 33/11 | 7/28 | 36 |
| <3 years | 40 | 36(0.90) | 4(0.10) | 31/5 | 9/25 | 35 |
| **Walking age** |  |  |  |  |  |  |
| ≥12 months | 62 | 60(0.97) | 6(0.10) | 48/12 | 11/41 | 54 |
| <12 months | 11 | 12(1.09) | 1(0.09) | 10/2 | 5/6 | 11 |
| **IQ** |  |  |  |  |  |  |
| <60 | 33 | 31(0.94) | 6(0.18) | 27/4 | 10/22 | 33 |
| ≥60 | 38 | 35(0.92) | 1(0.03) | 27/8 | 4/23 | 28 |
| **Language** |  |  |  |  |  |  |
| Non-verbal | 47 | 47(1.00) | 5(0.11) | 38/9 | 12/30 | 43 |
| verbal | 30 | 31(1.03) | 2(0.07) | 25/6 | 4/22 | 27 |
| **Serum 5-oxyproline** |  |  |  |  |  |  |
| decreased^$^ | 31 | 33(1.06) | 3(0.10) | 26/7 | 9/19 | 29 |
| non-decreased | 44 | 42(0.95) | 4(0.09) | 35/7 | 7/31 | 29 |
| **Carnitine (C0-C6)** |  |  |  |  |  |  |
| increased^$^ | 5 | 7(1.40) | 1(0.20) | 5/2 | 1/5 | 6 |
| decreased^$^ | 8 | 7(0.88) | 0(0.00) | 6/1 | 0/6 | 6 |
| normal | 62 | 61(0.98) | 6(0.10) | 50/11 | 15/39 | 56 |
| **Carnitine (C13-C18)** |  |  |  |  |  |  |
| increased | 52 | 48(0.92) | 4(0.08) | 37/11 | 9/30 | 41 |
| decreased | 3 | 6(2.00) | 0(0.00) | 5/1 | 0/5 | 5 |
| normal | 20 | 21(1.05) | 3(0.15) | 19/2 | 7/15 | 22 |
| **Serum thyroxin#** |  |  |  |  |  |  |
| increased | 16 | 3(0.19) | 1(0.06) | 3/0 | 3/1* | 4 |
| decreased | 4 | 3(0.75) | 1(0.25) | 3/0 | 3/1* | 4 |
| normal | 55 | 60(1.09) | 4(0.07) | 47/13 | 10/39 | 51 |

^ Potential harmful mutations include missense, loss of function and non-frameshift indels.

* P<0.05

$ Increased or decrease more than two folds as compared to the reference

# One patient had both serum increased FT3 and decreased FT4
